# Gender-Specific Factors Associated with Health-Related Quality of Life in Obese Korean Older Adults: Evidence from the 2020 Korean National Health and Nutrition Examination Survey

**DOI:** 10.3390/ijerph19148275

**Published:** 2022-07-06

**Authors:** Hee-jeong Kim, Dahye Park

**Affiliations:** 1 Department of Nursing, Namseoul University, 91, Daehak-ro, Seobuk-gu, Cheonan-si 31020, Korea; yshbb@nsu.ac.kr; 2 Department of Nursing, Semyung University, Semyungro 65, Jecheon-si 27136, Korea

**Keywords:** gender, health-related quality of life, obesity, older adults

## Abstract

Given the increasing aging population in South Korea, the quality of life of older adults must be ensured. This cross-sectional descriptive study investigated the gender-specific factors associated with health-related quality of life in obese older adults aged 65 years and above based on Korean National Health and Nutrition Examination Survey (KNHNES) 2020 data. In total, 507 obese Korean older adults participated in the 8th KNHNES. Chi-square tests and logistic regression analysis were performed to determine the variation in health-related quality of life according to socioeconomic and health-related factors and assess their inter-relationships. The influencing factors of health-related quality of life in obese Korean older adults were national health insurance (odds ratio (OR) = 1.02, 95% confidence interval (CI): 0.40–2.21), private health insurance (OR = 0.36, 95% CI: 0.28–0.75), arthritis (OR = 6.64, 95% CI: 2.57–17.14), and good dietary lifestyle (OR = 0.07, 95% CI: 0.05–0.93) in men; and private health insurance (OR = 2.66, 95% CI: 1.05–6.72), arthritis (OR = 2.81, 95% CI: 1.44–5.51), and physical activity (OR = 4.33, 95% CI: 1.71–10.94) affected health-related quality of life in women. The importance of health behaviors should be considered in the development of health programs and interventions for improving the quality of life of older adults.

## 1. Introduction

The rate of aging in South Korea has been the highest among the Organisation for Economic Co-operation and Development member countries in the past four decades. According to Statistics Korea, the percentage of Korean older adults aged 65 years and above was 14.9% in 2019 and was predicted to surpass Japan (36.7%), the most aged country globally, by 2045 [1]. With an increasing older adult population, the life expectancy has also markedly increased to 82.4 years, resulting in a lengthened average lifespan. Hence, the concept of health in senescence is not simply defined as life sustenance but directed at “healthy aging” or living a self-satisfactory healthy life. Interest in enhancing the quality of life beyond disease prevention and treatment has thus steadily increased [2]. Older adults are generally frail due to declining physical and mental capabilities, and therefore, health-related quality of life (HRQOL) is of major concern. However, it is possible to improve their quality of life (QOL) by investigating factors related to HRQOL.

Obesity is an important worldwide public health issue, which increases the risk of metabolic syndrome, diabetes, hypertension, dyslipidemia, and cardiovascular disease, irrespective of ethnicity, gender, and age [3,4]. Moreover, obese older adults have limited social activities due to impairments in activities of daily living and mobility that directly or indirectly affect their QOL [4]. Psychosocial factors also play a critical role in the treatment of diseases [5]. Notably, body mass index (BMI) in older adults is an important indicator of physical and psychological QOL in senescence [6,7] that can be used to evaluate the need to devise an appropriate weight management plan [8]. In particular, overweight older adults have lower HRQOL than older adults with average weight [8].

Previous studies have assessed socioeconomic factors such as education, economic status, residential area, current employment [9,10,11], smoking habit, and healthy lifestyle [10] that influence HRQOL, as well as health-related behaviors including regular exercise, sleep, frequency of alcohol drinking, muscle training, and walking [12,13,14]. There were also studies investigating HRQOL in older adults with a focus on individual health status, performance of daily activities, depression, and health-behaviors [10,15,16].

A comprehensive analysis of the variations across these factors may contribute to providing appropriate policies and resources for the individuals concerned. It is crucial for projects and policies regarding health promotion behaviors to extensively consider factors such as age, gender, socioeconomic status, and health-related and psychological characteristics. Hence, it is necessary to determine the subjective health perception in Korean older adults for health promotion. However, it is challenging to comprehensively investigate the entire population in South Korea. In this study, we specifically determined the HRQOL in male and female obese Korean older adults aged 65 years and above using the data of the 2020 Korean National Health and Nutrition Examination Survey (KNHNES), with verified reliability and representativeness regarding socioeconomic status and physical and mental health variables to provide the basic data for developing policies to improve health quality based on gender.

## 2. Materials and Methods

### 2.1. Study Design

This study involved a secondary analysis using data from the 8th KNHNES, targeting the nationwide population in South Korea in 2020. The correlations of HRQOL in obese Korean older adults with socioeconomic status and physical and mental health variables were analyzed based on gender.

### 2.2. Study Population

**Figure 1:**
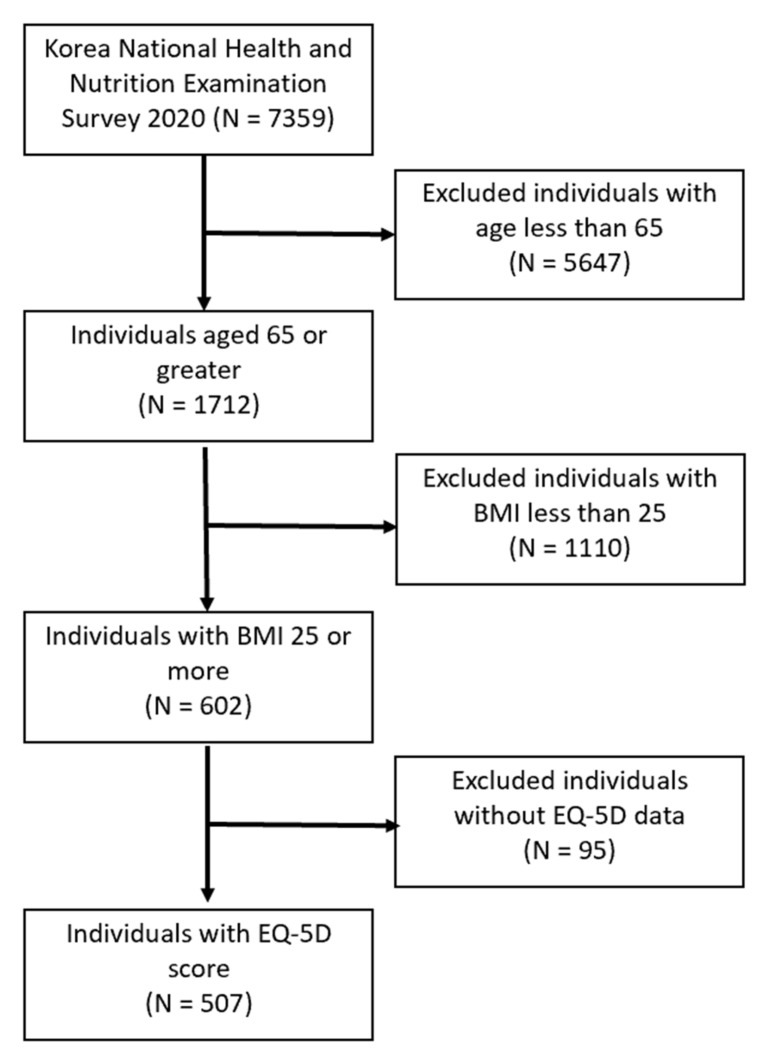
Flow diagram of the inclusion and exclusion criteria of the study. BMI, body mass index; EQ-5D, European Quality of Life-5 Dimensions.

The number of respondents to the KNHNES 2020 was 7359, with 1712 older adults aged 65 years and above. After excluding those without BMI data or with BMI less than 25 (*n* = 1110) and those without EuroQol-5 dimension (EQ-5D) data (*n* = 95), the number of participants included in this study was 507 (Figure 1). Height was measured to an accuracy of 0.1 cm while the participants were standing straight without shoes. Weight was measured to an accuracy of 0.1 kg while the participants wore a light garment. BMI was calculated by dividing the weight (kg) by height squared (m^2^). Thus, the obese group comprised those with BMI ≥ 25 kg/m^2^.

### 2.3. Instruments

#### 2.3.1. HRQOL

HRQOL was measured using a tool developed by Euroqol [17]. EQ-5D scores were measured using the self-report questionnaire. The tool consists of five subcategories: mobility (M), self-care, usual activities, pain/discomfort, and anxiety/depression. Each category is rated as no problems, some problems, and severe problems. The European Quality of Life-5 Dimensions (EQ-5D) index as the overall HRQOL score was calculated using an equation with weighted values. The highest score of one was obtained if all five subcategories were rated as no problems. Higher scores indicated higher HRQOL [17]. For the tool’s reliability, the intra-class correlation coefficient in the test–retest analysis was 0.65. The validity of the tool was confirmed based on previously known group validity and convergent and discriminant validity [18].

#### 2.3.2. Demographic Factors

Demographic factors comprised age, marital status, and total household members. Age was divided into three groups: <70 years, ≥70 years and <75 years, and ≥75 years. Marital status was divided into married (cohabitation) and unmarried (separated, bereaved, divorced, or unmarried) groups. Household was categorized as one-person (no cohabitant) and multi-person (one or more cohabitants) based on the response to the question “How many cohabitants are there in your household?”

#### 2.3.3. Socioeconomic Factors

Socioeconomic factors comprised family income, education, economic activity, home ownership, basic livelihood security, health insurance type, and private health insurance. Family income was categorized as high, middle-high, low-middle, and low based on the total mean monthly income quartiles. Education was categorized as elementary school, middle school, high school, and college graduates. Economic activity was categorized based on the response to the question “In the past week, have you worked for one or more hours to earn an income or been an unpaid family worker for 18 h or more?” as No (“Unemployed”) and Yes (“Employed”). Home ownership was categorized based on ownership of a house under the name of a household member as No (“None”) and Yes (“Have”). Basic livelihood security was categorized into Yes and No. Health insurance type included National Health Insurance and Health Care Coverage. Private health insurance was categorized based on the response to the question “Are you insured by any private health care insurance supporting the health care cost such as cancer insurance, cardiovascular disease insurance, and accident cover insurance?” as No (“None”) and Yes (“Have”). Unmet medical needs refer to the cases that did not receive necessary health care (examination or treatment) in the past year. The categories were based on the response as “I have had an unmet medical need” (“Have”) and “I have never needed a health care service” (“None”).

#### 2.3.4. Health-Related Factors

Health-related factors comprised alcohol drinking, smoking, hypertension, arthritis, inactivity, sleep, exercise, unmet medical needs, and dietary lifestyle. Alcohol drinking was divided into “High-risk drinkers”, defined as individuals drinking alcohol twice a week or more based on the Korea Disease Control and Prevention Agency (KDCPA) criteria (seven glasses or more for males and five glasses or more for females), and “Non-high-risk drinkers”, defined as individuals drinking with lower frequency and amounts than “high-risk drinkers”. Smoking was divided into “smoker” (current smokers consuming ≥ 5 packs a year) and “non-smoker” (past smokers and non-smokers). Hypertension and arthritis were defined as current cases diagnosed by physicians. Sleep duration was categorized based on the mean daily hours of sleep into groups of <5 h or ≥9 h and ≥5 h and <9 h. Exercise was analyzed based on the information of “a physical activity of a moderate level that makes you feel a little more fatigue than usual or a little out of breath” being performed for ≥30 min per session on five days or more a week, and of “a walk from one location to another” being performed for ≥30 min per session on five days or more per week. Annual weight change was categorized into “None”, “Decrease”, and “Increase”. Dietary lifestyle was categorized as “Good” if an adequate amount of food was available and as “Poor” if financial difficulties prevented an adequate supply of food. Suicidal ideation and mental health counseling were categorized as “None” and “Have” based on the corresponding questions. Stress was categorized based on the question on the level of stress on a typical day: “None” if the response was a negligible level and “Have” if the response was a very high, high, or low level.

### 2.4. Data Analysis

Collected data were analyzed using SPSS version 25.0 (IBM Corp., Armonk, NY, USA). The variations in socioeconomic status, health-related factors, and HRQOL in obese Korean older adults were analyzed according to gender using the χ^2^ test. A multivariate analysis was performed to determine the correlations of HRQOL with socioeconomic status and health-related factors in obese Korean older adults according to gender. For the multivariate analysis, the logistic regression that could incorporate the survey characteristics was used.

### 2.5. Ethical Considerations

The KNHNES was approved by the Institutional Review Board (IRB) of the KDCPA (IRB No. 2019-01-03-2C-A). The KNHNES raw data were available on the KDCPA website, data acquisition was approved, and the data were downloaded on 4 March 2022. The present study was exempt from approval by the IRB of the present institute (IRB No. SMU-EX-2022-04-003).

## 3. Results

### 3.1. HRQOL According to Demographic and Socioeconomic Factors of the Participants

A total of 224 men and 283 women were included to determine whether the HRQOL varied significantly according to the demographic and socioeconomic characteristics. Among the demographic factors, marital status had a significant effect on HRQOL in men (t = 5.11, *p* = 0.005). Meanwhile, among the socioeconomic factors, family income (F = 3.146, *p* = 0.026), health insurance type (t = 6.883, *p* = 0.007), private health insurance (t = 5.594, *p* = 0.022), and unmet medical needs (t = 3.678, *p* = 0.019) had a significant effect on HRQOL in men, whereas health insurance type (t = 2.515, *p* = 0.038) and private health insurance (t = 1.951, *p* = 0.003) had a significant effect on HRQOL in women (Table 1).

### 3.2. HRQOL According to Health-Related Factors (Lifestyle Behaviors)

For health-related factors, the HRQOL in males varied significantly according to arthritis (t = 1.780, *p* < 0.001), inactivity (t = 5.584, *p* < 0.001), annual weight change (F = 3.825, *p* = 0.023), dietary lifestyle (F = 3.106, *p* = 0.028), depression (t = 0.280, *p* < 0.001), mental health counseling (t = 3.475, *p* = 0.012) and stress (t = 1.083, *p* = 0.001). Meanwhile, the HRQOL in females varied significantly according to smoking habit (t = 9.703, *p* = 0.034), hypertension (t = 9.187, *p* = 0.019), arthritis (t = 0.180, *p* < 0.001), inactivity (t = 7.294, *p* < 0.001), sleep duration (t = 3.395, *p* = 0.032), moderate to intense exercise activity (t = 1.693, *p* = 0.004), exercise from one location to another (t = 7.770, *p* < 0.001), dietary lifestyle (t = 6.237, *p* = 0.002), depression (t = 5.379, *p* < 0.001), suicidal ideation (t = 1.976, *p* = 0.034), mental health counseling (t = 1.082, *p* = 0.001) and stress (t = 0.602, *p* = 0.001) (Table 2).

### 3.3. Correlation of HRQOL with Socioeconomic Status and Health-Related Factors

The significant differences in the result of logistic regression and regression coefficients were analyzed in two models (Table 3).

In Model 1, the correlations of HRQOL with socioeconomic factors were analyzed via logistic regression. The odds ratio (OR) for family income in men was 3.43 (95% CI = 1.30–9.04) for “high”, with “low” as the reference to indicate statistical significance. The OR for “have” private health insurance in men was 0.43 (95% CI = 0.22–0.82), with “none” as the reference to indicate significance. The OR for “have” private health insurance in women was 0.57 (95% CI = 0.34–0.95), with “none” as the reference to indicate significance.

In Model 2, the variables were controlled by adding health-related factors in Model 1. Regarding socioeconomic factors in men, the OR for national health insurance in health insurance type was 1.02 (95% CI = 0.40–2.21), with health care coverage as the reference to indicate statistical significance. Meanwhile, the OR for “have” private health insurance was 0.36 (95% CI = 0.28–0.75), with “none” as the reference to indicate significance. Regarding health-related factors in men, the OR for “have” arthritis was 6.64 (95% CI = 2.57–17.14), with “none” as the reference to indicate significance, and the OR for “good” dietary lifestyle was 0.07 (95% CI = 0.05–0.93), with “poor” as the reference to indicate significance. In socioeconomic factors for women, the OR for “middle-to-high” family income was 2.66 (95% CI = 1.05–6.72), with “low” as the reference to indicate significance, and the OR for “have” private health insurance was 0.49 (95% CI = 0.25–0.95), with “none” as the reference to indicate significance. In health-related factors for women, the OR for “have” arthritis was 2.81 (95% CI = 1.44–5.51), with “none” as the reference to indicate significance, and the OR for “have” inactivity was 4.33 (95% CI = 1.71–10.94), with “none” as the reference to indicate significance.

## 4. Discussion

This study determined the gender-specific differences in HRQOL in obese Korean older adults based on demographic, socioeconomic and health-related factors, as well as analyzed the correlation using the data from the KNHNES 2020.

The HRQOL of obese female Korean adults based on demographic factors was low in several cases, which corroborates the findings of Hong [19]. Compared with males, female older adults showed longer life expectancy and duration of living alone, for which an extended scope of social participation, training and opportunity of economic activity, and public health care policy seems necessary. Thus, promotion of a more optimistic gender-specific HRQOL should be performed.

Meanwhile, the HRQOL based on socioeconomic factors of obese male older adults varied according to family income, health insurance type, private health insurance, and unmet medical needs, whereas in women, the HRQOL varied solely based on private health insurance. The markedly high OR for having private health insurance leading to high HRQOL was observed in both males and females. This coincided with a previous study reporting higher HRQOL for those with higher family income [20]. Individuals with low family income had difficulties in accessing health information and services, preventing them from receiving health promotion and disease prevention services [20]. Economic strength in older adults had a positive effect on health, and a key influencing factor of HRQOL was private health insurance [21]. In addition, the level of unmet medical needs in older adults was high in those with a low socioeconomic status. This was similar to the findings of Hoebel et al., who reported that the burden of transportation and medical cost of using health care services resulted in the burden of addressing medical needs in older adults [22]. A low socioeconomic status signifies a deferred decision of using health care services due to insufficient allocation for health management. Such unmet medical needs could cause health inequality and, hence, it is necessary to develop measures to increase the accessibility of the health care system to older adults with a low socioeconomic status. Continuous self-management through health behaviors, such as periodic medical checkups, that improve the physical health of an individual could enhance the QOL and life expectancy in older adults, as well as reduce the caregiving burden on the family and the government [23]. Recent studies on the relationship between HRQOL and health status based on the socioeconomic status including ethnicity showed that various socioeconomic resources had a significant impact on health outcomes [24,25]. This is consistent with the present study, placing further emphasis on the health inequality in older adults with a low socioeconomic status as an important social issue given the rapidly growing aging population. This implies the need for basic and long-term measures for the health of the older adult population in the future.

Furthermore, while all older adults experience aging-related changes and constraints, the way obese older adults viewed their aging has been shown to affect self-satisfaction related to health and QOL [26]. Hence, the influence on the QOL may be attributed not to the practice of ineffective health behaviors but to whether an individual regarded his or her health-related problems as a natural part of aging or as a constraining disorder or disease in terms of comorbidity, inactivity, and physical activity. Considering that the recommended intake of proteins, vitamins, and minerals for older adults based on the KDRIs is not much lower than that for younger adults [27], the result of poor subjective perception on dietary lifestyle in older adults raises concerns. Community nurses should take particular care in providing interventions tailored to the individual needs of older adults to ensure regular and balanced nutritional intake. They should relay information on programs such as the delivery of meals and side dishes and free or covered services for food and restaurants for older adults, while assisting in nutritional diagnosis and counseling, as well as taking on economic and political roles in meal preparation. In addition, arthritis and inactivity were shown to affect the physical and psychological domains of QOL, respectively [28]. The QOL will be enhanced with programs that reinforce optimism or motivation in older adults to increase their positive self-perception of health and with various assistive tools to alleviate inactivity and information on social resources to increase mobility.

This study has certain limitations. First, this study combined and analyzed the data of the KNHNES 2020, which was conducted at the time of this study as a cross-sectional investigation of the relevant variables. As the data of a cross-sectional study restrict time-dependent before and after relationships, only the correlations, but not the cause–effect relations, could be determined between subjective health status and the socioeconomic factors, health-related factors, and psychological factors.

Second, the small sample size of the current data source implicated the presence of variables that could not be addressed in this study. If an adequate sample was ensured, we could have performed subgroup analyses according to age for identifying which age was more heavily influenced by obese factors. In addition, the monthly income in this study was based on the total mean monthly family income, where there was a possibility of dishonest responses; the resulting limitation was mitigated by applying an additional variable of health insurance type (e.g., national health insurance, health care coverage, no coverage) that could indicate the level of income. Moreover, missing values were found for approximately 30% of socioeconomic factors. Lastly, the health survey data used in this study were obtained through a self-reporting questionnaire, and thus there could have been certain bias in personal characteristics or circumstances in addition to the recall bias.

## 5. Conclusions

Variations in health behaviors were found in accordance with the analyzed factors, suggesting the need to incorporate such differences in the development of public health care policies and programs for obese older adults. As HRQOL exerts a significant effect on the health and mortality of obese older adults, various social efforts should be taken to ensure continuous monitoring of HRQOL and to support health services including health behaviors to enhance the HRQOL in obese older adults. Moreover, for public health care projects to reflect the ever-changing society, studies on the health behaviors that affect the HRQOL in obese older adults should be periodically conducted.

## Figures and Tables

**Table 1:** Difference in health-related quality of life by demographic and socioeconomic factors.

	Characteristics	Men			Women		
		*n*(%)	HRQOL		*n*(%)	HRQOL	
	N	224	Mean ± Standard Deviation	t or F (p)	283	Mean ± Standard Deviation	t or F (p)
Age, Years	<70 70 ≤ age <75 ≥75	97(43.3) 62(27.7) 65(29.0)	0.94 ± 0.12 0.93 ± 0.10 0.89 ± 0.13	2.97 (0.053)	117(41.3) 86(30.4) 80(28.3)	0.90 ± 0.13 0.88 ± 0.13 0.86 ± 0.14	2.28 (0.104)
Marital Experience	Unmarried Married	31(13.8) 193(86.2)	0.87 ± 0.15 0.93 ± 0.11	5.11 (0.005) *	125(44.2) 158(55.8)	0.87 ± 0.14 0.90 ± 0.14	0.003 (0.073)
Households	One-Person Multi-Person	33(14.7) 191(85.3)	0.89 ± 0.15 0.93 ± 0.11	4.67 (0.089)	83(29.3) 200(70.7)	0.88 ± 0.14 0.88 ± 0.14	0.022 (0.857)
Family income	Lowest Low-middle Middle-high High	48(21.4) 55(24.6) 63(28.1) 58(25.9)	0.89 ± 0.17 0.91 ± 0.10 0.95 ± 0.08 0.93 ± 0.10	3.146 (0.026) *	67(23.8) 76(27.0) 61(21.7) 77(27.4)	0.86 ± 0.14 0.90 ± 0.12 0.89 ± 0.02 0.88 ± 0.02	1.120 (0.341)
Education	≤Elementary Middle High school ≥College	80(35.9) 38(17.0) 66(29.6) 39(17.5)	0.90 ± 0.11 0.93 ± 0.12 0.93 ± 0.13 0.96 ± 0.10	1.998 (0.115)	186(66.2) 49(17.4) 35(12.5) 11(3.9)	0.87 ± 0.14 0.92 ± 0.10 0.92 ± 0.15 0.86 ± 0.06	2.571 (0.055)
Economic activity	Unemployed Employed	120 (103)	0.91 ± 0.13 0.94 ± 0.09	5.579 (0.069)	184 97	0.88 ± 0.14 0.88 ± 0.14	0.374 (0.818)
Home ownership	None Have	48 176	0.91 ± 0.15 0.93 ± 0.10	1.391 (0.312)	73 210	0.88 ± 0.13 0.88 ± 0.14	0.564 (0.833)
Basic livelihood security	None Have	208(92.9) 16(7.1)	0.93 ± 0.12 0.89 ± 0.11	0.331 (0.221)	247(87.3) 36(12.7)	0.89 ± 0.14 0.86 ± 0.14	0.013 (0.278)
Health insurance type	Health Care Coverage National Health Insurance	17(7.6) 207(82.4)	0.85 ± 0.17 0.93 ± 0.11	6.883 (0.007) *	29(10.2) 254(89.8)	0.83 ± 0.15 0.89 ± 0.13	2.515 (0.038) *
Private health insurance	None Have	84(37.7) 139(62.3)	0.90 ± 0.12 0.94 ± 0.11	5.594 (0.022) *	120(42.6) 162(57.4)	0.86 ± 0.15 0.90 ± 0.12	1.951 (0.003) *
Unmet medical needs	None Have	217(97.7) 5(2.3)	0.92 ± 0.11 0.80 ± 0.19	3.678 (0.019) *	259(92.2) 22(7.8)	0.88 ± 0.14 0.87 ± 0.14	1.046 (0.525)

HRQOL, health-related quality of life. * < 0.05.

**Table 2:** Difference in health-related quality of life by lifestyle habits.

	Characteristics	Men			Women		
		*n*(%)	HRQOL		*n*(%)	HRQOL	
		224	Mean ± Standard Deviation	t or F (p)	283	Mean ± Standard Deviation	t or F (p)
Alcohol	<High-risk drinking ≥High-risk drinking	19(8.5) 205(91.5)	0.94 ± 0.08 0.92 ± 0.12	2.182 (0.452)	96(33.9) 187(66.1)	0.89 ± 0.13 0.88 ± 0.14	0.499 (0.840)
Smoking	Never smoker, <5 packs years ≥5 packs years	56(25.0) 168(75.0)	0.92 ± 0.12 0.92 ± 0.12	0.001 (0.955)	274(96.8) 9(3.2)	0.89 ± 0.13 0.79 ± 0.24	9.703 (0.034) *
Hypertension	No Yes	84(37.5) 140(62.5)	0.93 ± 0.10 0.92 ± 0.13	0.779 (0.368)	103(36.4) 180(63.6)	0.91 ± 0.11 0.87 ± 0.15	9.187 (0.019) *
Arthritis	No Yes	182(81.3) 42(18.8)	0.94 ± 0.11 0.84 ± 0.12	1.780 (0.000) *	130(45.9) 153(54.1)	0.92 ± 0.13 0.86 ± 0.14	0.180 (0.000) *
Inactivity	No Yes	199(88.8) 25(11.2)	0.94 ± 0.10 0.81 ± 0.17	5.584 (0.000) *	238(84.1) 45(15.9)	0.91 ± 0.11 0.74 ± 0.16	7.294 (0.000) *
Sleep duration	≥5 h and <9 h <5 h or ≥9 h	23(10.3) 201(89.7)	0.91 ± 0.11 0.92 ± 0.12	0.565 (0.604)	43(15.2) 240(84.8)	0.84 ± 0.16 0.89 ± 0.13	3.395 (0.032) *
Exercise: moderate-intense activity	No Yes	215(96.0) 9(4.0)	0.92 ± 0.12 0.94 ± 0.10	0.142 (0.669)	278(98.2) 5(1.8)	0.89 ± 0.13 0.71 ± 0.20	1.693 (0.004) *
Exercise: one location to another	No Yes	119(53.1) 105(46.9)	0.92 ± 0.11 0.92 ± 0.12	1.091 (0.760)	140(49.5) 143(50.5)	0.85 ± 0.15 0.91 ± 0.11	7.770 (0.000) *
Annual weight change	None Increase Decrease	166 26 32	0.93 ± 0.11 0.94 ± 0.10 0.87 ± 0.14	3.825 (0.023) *	184 37 62	089 ± 0.13 0.87 ± 0.14 0.86 ± 0.14	1.316 (0.270)
Dietary lifestyle	Good Poor	167 7	0.93 ± 0.11 0.79 ± 0.11	3.106 (0.028) *	220 10	0.91 ± 0.11 0.80 ± 0.15	6.237 (0.002) *
Depression	None Have	213(95.1) 11(4.9)	0.93 ± 0.11 0.80 ± 0.13	0.280 (0.000) *	256(90.5) 27(9.5)	0.89 ± 0.13 0.78 ± 0.17	5.379 (0.000) *
Suicidal ideation	None Have	222(99.1) 2(0.9)	0.92 ± 0.12 0.91 ± 0.13	0.000 (0.862)	279(98.6) 4(1.4)	0.89 ± 0.14 0.74 ± 0.07	1.976 (0.034) *
Mental health counseling	None Have	221(98.7) 3(1.3)	0.92 ± 0.11 0.76 ± 0.21	3.475 (0.012) *	276(97.5) 7(2.5)	0.89 ± 0.13 0.76 ± 0.18	1.082 (0.016) *
Stress	None Have	201(89.7) 23(10.3)	0.93 ± 0.11 0.85 ± 0.17	1.083 (0.001) *	228(80.6) 55(19.4)	0.90 ± 0.13 0.83 ± 0.14	0.602 (0.001) *

HRQOL, health-related quality of life. * < 0.05.

**Table 3:** Odds ratios and 95% confidence interval for the associated factors with impaired health related quality of life (EuroQol-5 dimension index score) by gender.

Categories	Characteristics	Men				Women			
		Model 1		Model 2		Model 1		Model 2	
		OR	95% CI	OR	95% CI	OR	95% CI	OR	95% CI
Marital experience	Unmarried (Reference)								
	Married	0.56	0.23–1.38	1.00	0.50–1.48	0.82	0.49–1.36	0.76	0.39–1.47
Family income	Lowest (Reference)								
	Low-middle	1.00	0.42–2.39	0.75	0.48–1.89	0.81	0.41–1.61	1.02	0.41–2.57
	Middle-high	0.74	0.33–1.66	1.08	0.81–2.93	1.85	0.91–3.74	2.66	1.05–6.72 *
	High	3.43	1.30–9.04 *	5.70	0.84–3.17	1.05	0.52–2.11	0.93	0.39–2.21
Health insurance type	Health Care Coverage (Reference)								
	National Health Insurance	0.97	0.30–3.21	1.02	0.40–2.21	0.68	0.29–1.57	0.64	0.20–2.02
Private health insurance	No (Reference)								
	Yes	0.43	0.22–0.82 *	0.36	0.28–0.75 *	0.57	0.34–0.95 *	0.49	0.25–0.95 *
Unmet medical needs	No (Reference)								
	Yes	1.09	0.15–7.79	0.57	0.18–1.86	1.29	0.52–3.19	0.70	0.19–2.60
Smoking	≥5 packs/year (Reference)								
	Never, <5 packs/year			1.58	0.75–2.21			0.53	0.08–4.01
Hypertension	Yes (Reference)								
	No			0.62	0.59–1.57			1.04	0.53–2.03
Arthritis	Yes (Reference)								
	No			6.64	2.57–17.14 **			2.81	1.44–5.51 *
Inactivity	Yes (Reference)								
	No			2.75	0.75–10.11			4.33	1.71–10.94 *
Annual weight change	No (Reference)								
	Decrease			2.49	0.85–7.26			1.28	0.59–2.78
	Increase			2.19	0.44–10.84			2.00	0.65–6.17
Dietary lifestyle	Poor (Reference)								
	Good			0.07	0.05–0.93 *			0.22	0.04–1.25
Sleep duration	≥5 h and <9 h								
	<5 h or ≥9 h			0.70	0.18–2.72			0.85	0.33–2.22
Exercise: moderate-intense activity	No (Reference)								
	Yes			0.34	0.02–5.20			4.69	0.28–79.56
Exercise: one location to another	No (Reference)								
	Yes			0.65	0.29–1.49			0.55	0.29–1.03
Depression	Have (Reference)								
	No			2.55	0.23–28.02			2.03	0.59–6.96
Mental health counseling	Have (Reference)								
	No			0.70	0.07–6.98			1.67	0.10–27.59
Stress	Have (Reference)								
	No			1.57	0.43–5.76			2.01	0.85–4.73
N-2LL Wald^2^(dt)		0.093		0.157		0.103		0.204	
		1643.471		1440.430		1572.592		1389.661	
		4.850(8).773		6.303(8).613		5.768(8).233		17.318(8).057	

CI, confidence interval; OR, odds ratio. * < 0.05, ** < 0.01.

## Data Availability

Not applicable.

## References

[1] Statistics Korea (2019). Population Status and Prospects of the World and Korea in 2019.

[2] Statistics Korea (2017). Life Expectancy and Disability Adjusted Life Expectancy.

[3] Bornstein S.R., Ehrhart-Bornstein M., Wong M.L., Licinio J. (2008). Is the worldwide epidemic of obesity a communicable feature of globalization?. Exp. Clin. Endocrinol. Diabetes.

[4] Wang L., Crawford J.D., Reppermund S., Trollor J., Campbell L., Baune B.T., Sachdev P., Brodaty H., Samaras K., Smith E. (2018). Body mass index and waist circumference predict health-related quality of life, but not satisfaction with life, in the elderly. Qual. Life Res..

[5] Dey M., Gmel G., Mohler-Kuo M. (2013). Body mass index and health-related quality of life among young Swiss men. BMC Public Health.

[6] Yoo J., Yoo K. (2006). The influence of smoking and smoking cessation on body weight and exercise abilities. J. Coach. Dev..

[7] Yeom J., Kim J.K., Crimmins E.M. (2009). Factors associated with body mass index (BMI) among older adults: A comparison study of the US, Japan, and Korea. J. Korean Gerontol. Soc..

[8] Yan L.L., Daviglus M.L., Liu K., Pirzada A., Garside D.B., Schiffer L., Dyer A.R., Greenland P. (2004). BMI and health-related quality of life in adults 65 years and older. Obes. Res..

[9] Suh S.R., Kim M.H. (2014). Influencing factors on the health-related quality of life of older adults living alone. J. Korean Gerontol. Soc..

[10] Moon S. (2017). Gender differences in the impact of socioeconomic, health-related, and health behavioral factors on the health-related quality of life of the Korean elderly. J. Digit. Converg..

[11] Kim K.S. (2017). Effects of the health status and health behavior health-related quality of life of the elderly living alone and living with their families: Using data from the 2014 community health survey. J. Korean Acad. Community Health Nurs..

[12] Lee D.H. (2010). The effects of quality of life in the elderly’s health condition. J. Korean Gerontol. Soc..

[13] Kim S.Y., Sohn S. (2012). Factors related to health-related quality of life in rural elderly women. J. Korean Gerontol. Nurs..

[14] Kim H.S. (2017). Effect of pain, nutritional risk, loneliness, perceived health status on health-related quality of life in elderly women living alone. J. Korea Converg. Soc..

[15] Kim J.Y., Lee S.G., Lee S.K. (2010). The relationship between health behaviors, health status, activities of daily living and health-related quality of life in the elderly. J. Korean Gerontol. Soc..

[16] Yang S.O., Cho H.R., Lee S.H. (2014). A comparative study on influencing factors of health-related quality of life of the elderly in senior center by region: Focus on urban and rural areas. J. Digit. Converg..

[17] EuroQol (2018). EQ-5D User Guides.

[18] Lee S. (2011). Validity and Reliability Evaluation for EQ-5D in Korea.

[19] Hong J.Y. (2018). A study on sex-specific quality of life among the elderly aged 65 years or older. J. Korea Contents Assoc..

[20] Hong S.H. (2016). A study on the quality of life in older adults and the influencing factors. J. Korean Fam. Resour. Manag. Assoc..

[21] Kim J.G. (2012). An analysis of health inequality in older adults according to income status: Based on the EQ-5D. J. Korean Gerontol. Soc..

[22] Hoebel J., Rommel A., Schröder S.L., Fuchs J., Nowossadeck E., Lampert T. (2017). Socioeconomic inequalities in health and perceived unmet needs for healthcare among the elderly in Germany. Int. J. Environ. Res. Public Health.

[23] Chung K.H., Yeom J.H., Hwang N.H., Kim J.S., Lee G.Y., Oh S.H. (2013). Quality of life of middle-aged and older persons. Health Soc. Welf. Rev..

[24] Kahn J.R., Fazio E.M. (2005). Economic status over the life course and racial disparities in health. J. Gerontol. B Psychol. Sci. Soc. Sci..

[25] Koroukian S.M., Bakaki P.M., Raghavan D. (2012). Survival disparities by Medicaid status: An analysis of 8 cancers. Cancer.

[26] Low G., Molzahn A.E., Schopflocher D. (2013). Attitudes to aging mediate the relationship between older peoples’ subjective health and quality of life in 20 countries. Health Qual. Life Outcomes.

[27] Lee Y.H., Yim J.Y. (2014). An analysis of food expenditure according to aging. Korean J. Agric. Manag. Policy.

[28] Tüzün H., Aycan S., İlhan M.N. (2015). Impact of comorbidity and socioeconomic status on quality of life in patients with chronic diseases WHO attend primary health care centers. Cent. Eur. J. Public Health.

